# Notes on Medical Matters and Medical Men in Paris

**Published:** 1847-06

**Authors:** David W. Yandell

**Affiliations:** Louisville, Ky.; Paris


WESTERN JOURNAL

OF

MEDICINE AND SURGERY.

JUNE, 1847.

**Art. I.**—*Notes on Medical Matters and Medical Men in Paris.* By David W. Yandell, M.D., of Louisville, Ky.

Last week, M. Blandin, in one of his lectures in his amphitheatre at the Hôtel Dieu, spoke of the following case that had occurred in his female ward, which is so remarkable in many respects that I have concluded to give you a brief report of the lecture, to which I listened, as I do to all of his most practical and valuable lectures, with the greatest pleasure.

The subject, a woman sixty years of age, has for two years past had a crural hernia of the left side, which occasionally protruded, and as often returned, until five days ago, since which time there have been unequivocal signs of strangulation; eructations, vomitings, which presented this morning a fecal character; neither gaseous nor solid matter has been passed by the anus. The tumor is painful, and of a very intensely reddish violet hue.

Although the diagnosis, so far as regards a strangulated hernia, is very clear, it is by no means so easy to determine its exact composition. That the intestine is comprised, the intensity of the symptoms, the complete suppression of the stools, and the nature of the matter thrown up, leave no doubt. But whether there is not something else besides the intestine is a question which, in the present condition of things, could not be answered, the tumor being too much inflamed to admit of our establishing the absence or presence of the omentum. Happily, however, the clearing up of this point has no weight in the decision we must come to, and the knowledge of the actual condition of the hernia, while it is a thing of infinitely more importance, is more easily arrived at.

The surgeon began this inquiry by assuming that gangrene existed, a diagnosis which was founded, in the first place, upon the probable degree of constriction exercised by the ring. Under the influence of a hernia of two years' standing, the inguinal canal, composed of elements which yield much more readily, would have dilated;while the canal ring, on the contrary, composed of different materials admits of very slight extension; its posterior boundary, formed by the horizontal branch of the pelvis, offers the resistance of bone; its anterior side, represented by the most solid portion of the Fallopian ligament, and its internal side by Gimbernat's ligament, it is distended only with extreme difficulty; its external side, formed by the deep layer of the *fascia lata* is more extensible, though this is very decidedly rigid.

Secondly, the hernia had been in a state of strangulation for five days, a length of time more than sufficient for the development of gangrene in similar cases.

Finally, the tumor presented an intensely reddish violet color, and pressure made with the finger obtained crepitation, which in such cases is a manifest sign of gangrene. We could not suppose that the mortification was confined to the external coverings of the sac, for it always proceeds in this case from the hernia to its envelops, that is eccentrically. The intestine itself is evidently in a state of suppuration, and is either already broken or on the eve of breaking.

In a case of this kind what is the duty of the surgeon? Abandoned to itself a cure of the hernia may possibly be effected by the efforts of nature alone—adhesions being established at the ring, and an artificial anus being formed. A well known example of this spontaneous recovery occurred to J. L. Petit, who one day on entering a tavern recognised the odor of gangrene, and, learning from whence it proceeded, examined the patient, found a hernia in a state of gangrene, announced a happy termination, made some prescriptions to encourage the patient, and on his return the subject was out of danger. But such an issue is far too rare to be counted upon, and M. Blandin proceeded to perform the operation by forming a transversal fold with the integuments upon the middle of the tumor, and an incision parallel to the tumor and perpendicular to the fold, is made to the base of this latter. The subcutaneous cellular tissue is infiltrated with a blackish liquid exhaling the odor of gangrene; an abundant serum rendered it easy to recognise and penetrate the sac. The engorgement of the ligament embarrassing the examination of the hernia, a second incision was made perpendicular to the first. The small intestine was now carefully examined; it was of a deep brown color, its walls hypertrophied, and a single one of its convolutions constituted the hernia. The evidences of gangrene appearing incontestable, M. B. decided not to practice *unbridling*, but to open the intestine in order to establish an artificial anus. He now introduced a female sound into the extremity whose greater dilatation indicated it as the superior, but no matter, either gaseous, solid or liquid escaped by the instrument, which he allowed to remain, in the hope that the evacuation would be voluntarily established before the visit on the next morning; this, however, was not the case, the patient being found in almost the same condition in which she had been left the day before, the sound having given egress to no fecal matter.

What was to be done? Suspecting that perhaps the female sound had not been introduced sufficiently in front, and that its eyes had probably been stopped up by the strangulated part, a gum-elastic sound was substituted, and pushed deeply in. Thinking, on the other hand, that the female sound might have missed its end, and entered the inferior instead of the superior extremity of the intestine, a second gum-elastic sound was introduced into the other, that which might be the inferior or perhaps the superior extremity. Still nothing escaped, either through these tubes or along their sides, which was equally the case the following day.

At the visit on the second day after the operation the patient seemed to be no worse; she even asked for food, and there were no appreciable signs of peritonitis. But the vomiting had continued and the tympanitis had augmented to such a degree, that the convolutions of the intestines were delineated almost plainly enough to be counted through the parietes.

On the supposition that the spasmodic contraction of the inferior end of the intestine had applied it on the sound and closed its eyes, or that some unforeseen obstacle, which it would be impossible to remove without a new operation, was opposed to the re-establishment of the course of the fecal matter, the following operation was determined upon. It was to consist of an incision which should admit of the two extremities of the intestine being drawn out, in order the better to submit them to examination by the sight and touch, and thus attain a more exact apprehension of the state in which they were. For the sake of preserving useful adhesions and preventing contraction of the intestine into the abdomen, which would have been consequent upon its escape from the artificial ligatures which held it in place, the incision made was from without inwards, as M. B. has already done more than once with success. The external coverings of Gimbernat's ligament were necessarily divided; the ligament itself was divided with the care necessary in proceeding from the superficial to the deep seated parts. The two ends of the intestine after detaching some slight adhesions, were drawn out to the length of about two centimetres. To our great astonishment, remarks M. Blandin, there was no expulsion of fecal matters. "A sound is placed in the superior extremity, nothing; another in the inferior, nothing still. In despair we left the two sounds until the next morning, after having prescribed a purgative injection; but the result was the same; nothing was discharged, and the patient died in the evening of the third day from the operation, and the eighth since the strangulation."

M. Blandin was utterly at a loss for a plausible explanation of this suspension of the course of the fecal matter, so prolonged, so obstinate, notwithstanding the opening of the intestine, notwithstanding the relief of the strangulation, the situation of the two extremities at the exterior, and the permanent presence in their cavities of a sound. A fecal clot clogging the small intestine, an internal strangulation, a volvulus, all seemed improbable.

The opening of the body, far from putting an end to our astonishment, M. Blandin continues, rather increased it.—"What obstacle did we find? None! The intestine was throughout perfectly healthy, and had preserved its proper calibre, and either of these operations that we performed would, in ordinary cases, have saved the life of the patient. If we could have obtained evacuations we should without doubt have prevented the development of the peritonitis which was the cause of the patient's death." This supposition seems so much the more likely as the inflammation of the serous membrane was slight, only characterised by some ounces of turbid liquid in the inferior pelvis, and some faintly developed false membrane around the ring.

M. Blandin was unwilling to admit, although he confessed he could not otherwise understand the phenomenon, either that the intestine had spasmodically contracted on and closed the eyes of the sound, or that clots of fecal matter had obstructed them. He closed by professing his intention hereafter to employ in such cases a more voluminous sound, pierced with a single eye at its extremity in the direction of its axis, as he believes that through an instrument of this kind the fecal matter would pass more easily.

The following very simple and easy method of detecting the presence of sugar in the urine of diabetic patients is practised in the Paris hospitals: Pour a small quantity of the urine into a glass tube, to which you add a few drops of the aqueous solution of the sulphate of copper, the tube being held in the flame of an alcohol lamp until the liquid boils. If the urine is healthy it produces no action on the salts of copper, and the liquid preserves the beautiful blue color which has been imparted to it by the copper; but if the urine contain sugar, this effects the decomposition of the salt, and as soon as ebullition takes place the mixture is observed to pass first to a green, afterwards to a fawn color, which is more deeply marked as the proportion of the sugar is greater. Liquid caustic potash may be substituted for the sulphate of copper, in which case the color of the mixture varies according to the amount of sugar present, from a yellow to a more or less deep brown.

In the minds of many practitioners the efficacy of iodine injections in the radical cure of hydrocele is still a question, and must remain so until still further testimony is added to that already on hand, which is not a little. A case occurred not long since at *Du Midi* hospital, in the service of Vidal, which I think may be regarded as almost unequivocally favorable to the value of iodine, and such, as will presently appear, was also the opinion of Vidal himself.

The patient was 43 years old, a house painter, who, about a year previous to his admission into the hospital, noticed for the first time an abnormal volume of his right testis. The organ gradually increased to the size of a man's fist, fluctuated, was regular, and offered the characteristic transparency of hydrocele. Some eight months after the appearance of the affection the patient presented himself to Vidal, who punctured the tumor and injected into the tunica vaginalis a little common wine in which had been boiled some Provins' roses. In ten days the serum re-accumulated in the vaginal tunic, formed a tumor almost as voluminous, and possessing the same characters as the first, especially the very marked transparency. Vidal again punctured it—and, with the view of more successfully irritating the tunica vaginalis, added this time a third of alcohol to the wine which he used for the injection. But in this attempt he was as unsuccessful as he had been in the former, for the hydrocele was re-formed and acquired in eight days the same volume, and in eleven days the same transparency.

The patient returned to the hospital on the 10th of December in order again to submit to the puncture and injection of the tumor, which latter was this time performed with the tincture of iodine (equal parts of water and tincture of iodine.) I should not omit to mention that this and the previous injections were made twice, and allowed to remain ten minutes, or thereabouts, in the vaginal tunic. The tumor was reproduced in a few days, diminished, somewhat, however, in volume, and remained stationary during the greater part of a month. A third relapse might have been anticipated, but the tumor presented none of the characters of the former; it was hard, unequal, sufficiently heavy to embarrass the patient, and not at all transparent. Vidal declared that a cure had been effected, but a slow one which he must aid, so on the 14th of January a vesicatory was placed on the right side of the scrotum. On the 18th, although the tumor presented some of the characters remarked previously to the application of the blister, it had sensibly diminished, and become somewhat painful. The blister was allowed to dry. The tumor had diminished considerably on the 21st, when Vidal ordered it to be covered with a coat of mercurial ointment in order to hasten resolution. The 28th, the volume of the two organs is the same, and the right, which hitherto has been painful, is now so no longer. On the 30th the testes have returned completely to their healthy state. This, although a tardy, is nevertheless a perfect cure, and it may, in my opinion, be fairly attributed to the influence of the iodine. I may remark that it agrees, in many respects, with the cases of slow recovery cited by Velpeau when on the subject of iodine injections in the surgical treatment of hydrocele.

The following curious case occurred not a great while since in the wards of M. Roux, at the Hôtel Dieu. The patient was a very robust man, 45 years of age, who has suffered for a month past from pains in the head, weakness in the legs, and general languor. Some days since his physician ordered him to apply twenty leeches to his anus, which was done, and immediately after their removal the perineum and scrotum became painful, red, and apparently erysipelatous. The pain and redness soon gained the abdominal parietes, and on the fifth day the abdomen was tense, red in its two inferior thirds, and seemed occupied by a vast phlegmonous abscess, and the scrotum itself appeared to be the seat of the same affection. Besides this there appeared to be a blenorrhœa of five or six years' standing; though he has never had any difficulty in urinating, and the jet has always been sufficiently large; only for the last two or three days he has had frequent desire to micturate, which, when done, is followed by very violent burning sensations in the urethra. A large catheter was introduced into the bladder with facility and gave issue to a large quantity of urine, notwithstanding the patient had urinated not an hour before. From what does this phlegmonous condition of the abdomen, accompanied by the most distressing pains, proceed? Is it an erysipelas occasioned by the bites of poisonous leeches? After an attentive and very minute examination, a severe palpitation of the diseased parts of the scrotum and perineum induced M. Roux to believe that there was urinous infiltration; and without delay large oblique incisions, from without inwards and from above downwards, were made upon the two sides of the abdomen; another, parallel to the median line of about twelve or fifteen millimetres in depth, and five or six centimetres in length; finally, a fourth was made from the top to the base of the scrotum. These incisions were really frightful, as well from their extent, as their depth, and situation. It seemed for sometime as though the testicle might escape and the intestines protrude through these different openings. But none of the serous cavities had been penetrated; neither the tunica vaginalis nor peritoneum had been injured, and the wounds gave exit only to a very large quantity of blood, mixed with a fetid ammoniacal urine.

The diagnosis of this rather remarkable case was rendered doubly difficult, by the absence of every known and appreciable cause with which we are able to connect this urinous infiltration. There would have been infinitely less embarrassment if the patient had been affected with stricture of the urethra, paralysis of the bladder, etc. Although the patient declares that he never attempted catheterism, there is no certainty that such efforts have not been made, perhaps by himself, and which have resulted in the formation of a false passage, which was the habitual cause of the retention of the urine.

In July, 1845, in a paper communicated to the Academy of Sciences M. Jobert (de Lambaille) has described a new method of operation which he had substituted for the one called *élytroplastic*, and which he designates *autoplastic par glassement;* at the same time he reported a case of vesico-vaginal fistula, occupying the whole extent of the urethra and of the vesico-vaginal septum, to about half a centimetre from the neck of the uterus, which he had completely cured by this operation, and which I proceed to describe, illustrated by the history of one or two cases.

*Vesico-vaginal fistula seated at the left side of the vagina directed from before backwards, at least six centimetres in length, complicated with loss of substance, separation of the borders of the fistula, and hernia of the bladder.—Approximation of the lips of the wound by interrupted sutures.—Lateral incision comprehending the whole thickness of the walls of the vagina, directed from behind forwards, and practised on the side opposite the fistula.*

Case I. — A woman, aged 22 years, entered the Saint Louis hospital on the 26th of June, 1846, to be treated for vesico-vaginal fistula supervening upon a tedious and difficult accouchment. Of a strong constitution, this young woman had never had any serious disease. She was married in her twentieth year, and gave birth to a child the year following, (September, 1845). The labor lasted eight days, and, proceeding with great slowness, the accoucheur was not called until the fourth day after the first pains had appeared. Every endeavor that he made to accelerate it was ineffectual, and on the eighth day he decided to terminate the labor by performing the operation of cephalotripsy. For three days the patient had not urinated.

After the delivery, intense pains were felt in the vesical and hypogastric regions. During the first eight days no difficulty was experienced in the passage of the urine. At the end of this time while the patient was turning herself in bed she felt a foreign body, of a dark color, about the size and dimensions of an almond, suddenly escape by the vulva, and at the same instant her bed was inundated with a gush of liquid, which from that moment has not ceased to flow. After the fall of the eschar, and the formation of the vesico-vaginal fistula, the strength, vital force, and *embonpoint*, even, soon returned, and in the space of a few weeks the woman was in full convalescence.

Notwithstanding the continual flow of urine it did not prevent her from resuming her occupation, and having in vain edeavored to mitigate her infirmity, she came to Paris and entered the Cochin hospital in the service of M. Michon; here she remained for three months, during which time, although she underwent many cauterizations with the red iron and nitrate of silver, no amelioration was obtained in the state of the fistula, and the patient, demanding to be dismissed, came to the hospital Saint Louis, when the following symptoms were observed: There no longer existed any disposition to urinate; the urine which had ceased to pass by the urethra, fell, involuntarily, drop by drop, into the interior to pass afterwards to the exterior in a continuous manner, whatever the position assumed by the patient. The index finger, introduced into the vagina a certain depth encountered, on the anterior wall of this canal, a large opening by which it easily penetrated into the bladder. Upon a careful examination of the diseased parts proceeding from without inwards, about the vulva, the perineum, and particularly on the internal and superior part of the thighs, a multitude of small, reddish tubercles were observed, hard and irregular, which seemed to be developed at these points entirely by the prolonged contact of the urine with the integuments. Besides this eruption, all of the skin contiguous to the genital organs was red, sensible, and hard to the touch; it was deprived of the epidermis in many points, and the urine which was continually discharged upon its surface produced smartings and very painful itchings. Except this, the genital organs presented nothing particularly noticeable.

The neck of the uterus examined with the speculum is normal. The fistula is situated on the anterior wall of the vagina, four centimetres behind the *meatus urinarius*, about five millimetres to the left of the median line, and is of a longitudinal form. Its dimensions in this course are more than six centimetres in extent, and its length occupies the greater part of the fundus of the bladder in such a way that it almost reaches the neck of the uterus. Its transverse diameter amounts to some centimetres; its borders are regular and seem as if cut; they are in a state of considerable and permanent distension; the orifice of the fistula remains open, and a portion of the bladder forms a hernia through this opening. The female sound introduced by the canal of the urethra, traverses the bladder, arises in the vagina, and may be brought back to the vulva if a see-saw movement is made.

As to the general state, it is satisfactory. The patient ardently desires the operation for which she has been for some days preparing, by the use of baths, injections, and clysters.

On the 2d of July, M. Jobert performed the operation in the following manner: Except the securing of the hands and feet together, the patient was placed in a position similar to that pursued in the operation for stone. The speculum with one valve was now introduced into the vagina, so as to depress its posterior wall; two assistants separated at the same time the large and small lips; this allowed the operator to perceive the neck of the uterus, and seizing it with the forceps of Museux he drew it outwards by light and moderate traction. The neck of the uterus in being displaced has drawn after it the fundus of the bladder, and consequently the fistula; this, presenting itself now at the entry of the vagina, became more accessible to the action of the cutting instruments. The borders were pared with the scissors and bistoury. This was the second step of the operation. A curved needle, armed with a double thread well waxed, was firmly fixed at the extremity of a needle bearer, and the left border of the fistula, being seized with a pair of forceps, was traversed by the needle from without inwards; this, deprived of its support, was brought back by the orifice of the fistula into the vagina. The needle being fixed to the extremity of the instrument was again introduced into the bladder, and penetrated from within outwards the corresponding border of the fistulous orifice; it was afterwards drawn into the vagina bringing after it the thread with which it was armed. This thread was thus disposed in the form of a loop to the inferior cavity in the thickness of the inferior lips of the fistula, in two corresponding points and about two lines from their inferior border. The two extremities of the thread were afterwards drawn out and maintained at the exterior, whilst, proceeding always from behind forwards, five other threads were successively passed through the corresponding borders of the fistula. All these threads were placed sufficiently near one another to prevent any free space from existing between them after the lips of the opening were brought together. After having withdrawn the sound from the bladder an incision, five centimetres in length, was made upon the right side of the vagina and the part of this canal which corresponded to the fistula. This incision immediately relieved the tension of its lips and allowed them to be readily brought in contact. M. Jobert now seized the first thread, and drawing lightly upon the two ends, the borders of the fistula came exactly in contact. A double knot was made to maintain them in this position, when the two ends of the thread were cut, in order, so to speak, to smooth the knot. The four remaining threads were tied, and afterwards cut in precisely the same manner.

The neck of the uterus, being freed from the hooks, returned immediately to the bottom of the vagina, which was repeatedly injected with cold water, and the operation was completed by the introduction of a light tampon consisting of a cylinder of agaric (*Polyporus igniarius*). The patient was carried to her bed with a sound to remain permanently placed in the bladder. Infusion of tilia (*tilia Europa*) to be given in a draught, with one ounce of syrup of poppy. During the day the patient had very little pain; but during the night she had not a moment of repose. The urine has passed perfectly well by the sound.

The 3d of July, a little fever; pulse at 80; skin hot; considerable thirst. The urine continues to pass wholly by the sound. Tilia, antispasmodic syrup of gum.

4th. The fever no longer exists; the urine has flowed by the sound, nevertheless the cloths placed under the seat of the disease are wet in some points.

5th. The sound was obstructed during the night and a quantity of urine was found under the patient; but upon the introduction of a new sound the urine resumed its usual course.

6th. The sound gave passage to the urine; the same was the case the days following; nevertheless the patient complained more than once that she felt the urine drop upon herself, and in the morning when it was changed the cloth was found wet.

From the 15th to the 24th of July, the urine passed almost entirely by the sound. The patient, moreover, had an appetite, tranquil sleep, and appeared to have entirely recovered from the operation.

On the 24th she was examined; one of the threads had fallen upon the posterior wall of the vagina; the others were cut with the scissors and drawn out by the forceps. When the finger was introduced into the vagina it was ascertained that the union of the fistula was complete. During the day the sound was not allowed to remain in the bladder; the patient was sounded very frequently, and every time nearly a glassful of urine was obtained.

The 25th, the sound was introduced and allowed to remain, and almost the whole of the urine passed through it.

The 29th, the patient was again examined, and the last portion of the thread removed from the vagina.

The 30th and 31st, a small granulation, seated in one of the points of the suture, was cauterised; the sound allowed passage to the whole of the urine.

August 5th. The patient was examined anew; the cicatrization was complete.

8th. The union seemed permanent.

13th. The sound was withdrawn. The next day the patient declared that she had micturated at will, and been able to retain her urine for quite a long time, but that nevertheless, there was a slight exudation into the vagina. On examination of the parts, nothing was discovered save a little point, almost imperceptible, situated at the posterior extremity of the cicatrix. This was touched with the nitrate of silver.

From the 14th to the 21st the sound remained, to be finally withdrawn on the 21st.

On the 24th, the patient affirmed that not a single drop of urine had flowed by the vagina. When the interior of the vagina was examined with the speculum, there was found upon the right lateral wall the trace of the incision made at this point. A regular cicatrix at the place of the fistula was observed on the exterior wall; the cicatrix was distinguished from the surrounding tissues by reddish discolorations, and by slight irregularities existing at the points of the suture. The genital organs are perfectly healthy. The general health is most satisfactory.

The 27th December, the patient does not lose a single drop of urine, and is able during many hours' succession to retain a very considerable quantity of urine in the bladder. The disposition to urinate is felt less frequently during the night than the day.

The examination with the speculum reveals only the smallest trace of the ancient fistula; but to the left and right of the median line two perfectly white cicatrices are perceived, which are directed from behind forward. Along the course of the cicatrix of the left side, white depressions corresponding to the points of the suture, are observed. The anterior wall of the vagina has diminished very little in size. The orifice of the urethra has almost regained its natural calibre, and the abnormal granulations that it presented has yielded to a few cauterizations with the nitrate of silver.

Finally, the patient has recovered her cheerfulness, bloom, and *embonpoint.*

*Vesico-vaginal fistula of the fundus of the bladder.—Autoplastic operation.—Recovery.*

Case II.—A woman, 24 years of age, and of strong constitution, entered Saint Louis hospital on the 3d of December, 1845. She had menstruated at the age of fifteen years, and married at twenty. Her first child had been extracted by the forceps, three days of pain not sufficing to effect its natural expulsion. She became pregnant a second time, was brought to bed at the full period, and as the labor was not more successful than the former had been, the cranium of the child was perforated, and the delivery effected by means of instruments. The operation occupied almost two hours, and was followed by violent inflammation of the genital organs. The first days after the accouchement offered nothing particular; the urine was evacuated voluntarily, and did not, in fact, pass by the vagina until after the lapse of six days. It passed at first only in part by the fistula, the reason of which was that the eschar was only partially separated, for as soon as it became wholly detached, the urine passed altogether by this opening. From this period the urine escaped involuntarily and incessantly, whatever might be the position of the patient.

The following was her condition on admission into the hospital.

1st. The genitals are constantly bathed with urine.

2d. All the parts with which the urine was in contact were erythematous, red, or tuberculous.

3d. The vesico-vaginal wall was irregular, unequal, and furrowed in a way which denoted the existence of ancient losses of substance, and cicatrized ulcerations.

4th. The neck of the uterus was irregular and ulcerated.

5th. In the track of the urine calculous deposits were found.

6th. In front of the neck of the uterus the touch detected a depression where the vesico-vaginal wall appeared manifestly diminished in thickness.

7th. The introduction of a liquid into the bladder immediately revealed the site of the fistula, for as soon as it entered the bladder it escaped by the vagina in the form of a continued jet.

8th. The introduction of the speculum presented to the eye a hard depression formed by the inodular tissue, and at the bottom of which a large sound may be passed directly into the bladder. The instrument passed along the neck of the uterus, which seemed grooved in order to assist in the formation of the fistula. While the patient sits up she is able to retain a small quantity of urine in the bladder.

9th. The patient is irritable, agitated, and the intellectual faculties sensibly disturbed. This is not the first woman in whom we have seen grave changes occur in the functions of the nervous system, on account of serious lesions of the bladder.

10th. When the patient coughs the urine escapes by jets. The direction and extent of the fistula having been ascertained, and an operation being judged indispensable, M. Jobert prepared the patient by baths, emollient and narcotic injections, and purgatives. When in the proper condition, he practised, on 23d of December, the autoplastic operation in the following manner:

The woman was placed upon a bed, the legs flexed upon the thighs, and the thighs upon the pelvis. The speculum with two valves was introduced and the neck of the uterus drawn to the exterior by means of Museux's forceps; then Jobert pared away from both the interior of the fistulous channel and the vaginal circumference of the fistula all the parts which seemed indurated. The vagina was afterwards detached from its connexion with the neck, when it immediately became easy to apply three points of the interrupted suture in order to maintain the lips of the fistula in contact. The bleeding surfaces were thus brought together without any difficulty, and without the slightest distension.

A sound was afterwards placed in the bladder, and an oat-chaff cushion under the knees.

On the day of the operation a sanguinolent urine first passed by the sound, but soon became clear and transparent. There was a little vesical tenesmus for sometime after the operation, but the pulse remained at 70.

On the 24th, the patient was allowed some broth; nothing particular was observed.

25th. A slight vesical tenesmus reappeared, but there was no fever nor any untoward symptom. In the afternoon she withdrew the sound and evacuated her urine spontaneously.

26th. The urine flowed by the sound alone until in the afternoon, when the patient withdrew and afterwards returned it without experiencing any inconvenience.

28th. The patient was not more manageable than she had been the preceding days, but urinated in part by the canal and in part by the sound.

Until the 11th of January she at times allowed the sound to remain, and at times withdrew it, and notwithstanding all these indiscretions, as we shall see in the end, she was completely cured.

The threads had been permitted to remain, and it was not until the 11th of January that the neck of the uterus was examined previously to their removal. The urine at this time (11th January) no longer passed by the fistula, but escaped in a continued jet from its reservoir whenever the patient endeavored to urinate, and she was able to retain a large quantity for a considerable length of time. When the speculum was introduced one of the threads used in the suture was discovered, and removed with the forceps.

January 15th. The patient had frequent desire to micturate; the speculum being again introduced, another thread was found and withdrawn. In the course of the following days the irritability of the bladder disappeared; all the urine was passed by the urethra.

Until the 6th of February everything progressed in the most favorable manner, notwithstanding the indocility of the patient, who had been over and over again guilty of imprudences, quite sufficient to compromise the cure of the fistula. She was on the point of being sent to her family, when, on the night of the 7th, she was seized with a well-marked paroxysm of mania. At the visit the next day, she was found in a state of considerable agitation; she did not utter a single word; the face was animated; eyes haggard; pulse accelerated. [Venesection; twenty leeches upon the mastoid processes; ice to the head.]

The maniacal condition continues; the patient sings, and is continually endeavoring to escape; great incoherence of ideas. [The straight-jacket was applied; fifteen leeches behind the ears; antispasmodic draught; vaginal injections.] From this time until the 12th of March she has almost uniformly refused to reply to the questions that have been put to her, and although the restlessness has been less, her reason is constantly disordered. New bleedings have been practised, many blisters applied to the neck and the mastoid processes, without producing any notable amelioration in the condition of the intellectual faculties. In this state the patient was removed on the 12th of March to a lunatic hospital. The examination with the speculum revealed the final result of the operation, which the patient had undergone. The neck of the uterus being seized and drawn towards the vulva, the point of the bladder which had been occupied by the fistula was easily recognized; there remained in its place a linear cicatrix a little depressed, a centimetre and a half, or thereabouts in extent, very solid and in every respect perfect, no fistulous opening remaining on its surface. When pressed with a compress or pair of forceps, it remained dry, furnishing not a drop of urine. The cure of the fistula was radical and complete.

Case III—relates to a Creole woman, aged 35 years, who had labored for ten years under a vesico-vaginal fistula with loss of substance, for which two operations had already been attempted without success. The fistula consisted of an enormous transversal slit about thirty-seven centimetres in size seated at the fundus of the bladder, entirely behind the level of the neck of the uterus, in such a way that the latter organ formed, in some manner, the posterior lip. The borders were hard and callous, the neck of the uterus itself diseased, ulcerated, and softened; the vagina indurated, painful, and presenting in various points calculous deposits, notwithstanding the incessant care and attention to cleanliness on the part of the patient.

M. Jobert operated on this patient on the 21st of October, 1846, in the presence of Professors Begin, Bouillaud, and others. The circumstances attending, and the phenomena which followed the operation are embodied in the preceding cases, it only remaining to be added here that there was no traumatic accident, and that from the time of the operation not a single drop of urine passed by the fistula, but the whole of it was discharged by the urethra, which had not given passage to it before for ten years. The first of the threads was removed on the 1st of November, when the adhesion of the lips of the wound was perfect, and on the 5th the union was already very solid.

On the 11th, the sound was withdrawn, but was introduced every two hours, and in the intervals the urine accumulated in the bladder; sometimes a few drops escaped by the urethra, but none passed by the fistula.

On the 22d, the patient urinated without assistance, and was able to retain the urine.

27th. The menses, suppressed for the last ten years, and replaced by an anal hemorrhage, were re-established, and on the 5th of December, when the patient was examined by Professors Begin, Bouillaud, &c., she was perfectly cured, and on the 10th set out for Marseilles.

Vesico-vaginal fistulæ, and particularly those affecting the fundus of the bladder, are among the most frightful infirmities to which the female is liable after difficult labors, and, when of considerable extent, are even now regarded by many of the most eminent writers in the profession, as beyond the reach of surgical art. But from these cases of M. Jobert we are justified in forming a more favorable prognosis. This surgeon stands deservidly high in France, and his reports may be confided in for accuracy and fidelity. His operations unquestionably have a title to a very high rank among the achievements of modern surgery. He promises at a future meeting of the Academy of Sciences to give the physiological and pathological reflections suggested by these cases.

While Germany has given to war, in the past year, another element of destruction in the cotton-powder; and France has assigned to Neptune, the fabled god of the seas, a place among the stars, America has made a richer offering to humanity in disclosing the precious virtues of the vapors of sulphuric ether. If the conquest of a new agent of attack and defence, if the discovery of a star, before unknown in the firmament, creates so lively an interest in the mind of every one, what should be the sensations caused by the application of an agent through whose mysterious powers the human constitution is made insensible to the pain attending surgical operations?

To Jobert belongs the merit of having made the first attempt, in France, to intoxicate with the vapors of sulphuric ether; his subject was a patient from whom he wished to remove a cancerous tumor of the lip. But from the defective apparatus for its administration, and some other unfavorable circumstances, his patient, though a man 59 years old, and though evidently affected, was not brought sufficiently under the influence of the ether to lose his sensibility, and thus the first experiment was considered as rather contradictory of the reports from the other side of the channel, where, at that time, the power of the ether to allay pain in surgical operations was verified every day. Malgaigne, also of St. Louis hospital, was the first French surgeon who succeeded in producing, by the gas, a degree of insensibility sufficiently great to destroy the pain of an operation. To the Academy of Medicine at its session on the 12th of January, the last named gentleman communicated the history of five cases in which he had used the ether, of which the following is a detailed account.

I. A young man, aged 18 years, having a suppurating phlegmon at the inferior part of the leg, was made to inhale the sulphuric ether for ten minutes, which sufficed to plunge him in a complete lethargy or sleep, during which the abscess was opened with a bistoury. The patient raised up half a minute after, declaring that he had felt nothing, and he did not even believe that he had undergone the operation.

II. An Italian, somewhat older, affected with a tumor of the neck, breathed the ether for five minutes; after returning to himself he said that, although he had been conscious that the tumor was being removed, he experienced no pain.

III. A young woman presenting also a tumor of the neck, lost consciousness after inhaling the ether for ten minutes, and although she did not feel the first incision, she recovered immediately after and suffered during the remainder of the operation as much as if she had not inhaled the ether.

IV. A man who had had his leg crushed by a railroad car passing over it, before submitting to its amputation, inhaled the ether for seventeen minutes, when lethargy being produced the limb was removed, and after he had recovered from his stupor, he declared that notwithstanding he had been conscious throughout of the removal of the limb, he had felt no more pain than if it had been slightly scratched with the point of a pocket knife.

The fifth and last operation was for strabismus in a young man; he breathed the ether for ten minutes without its seeming at all to affect his constitution, when he relinquished the attempt, and, of course, suffered during the operation as would a patient under ordinary circumstances.

Professor Roux obtained, a few days after, an incomplete result in a man aged 45 years, who had fallen on the pavement and broken his leg, which, from the severe consecutive lesions, finally required amputation. The patient inhaled the ether for twenty minutes, at the end of half of which time his eyelids fell, but he still answered questions.— The operation occupied twenty minutes, and I am inclined to believe that the man, in whom the pain was notably diminished, would not have cried out had not the students from all parts of the theatre repeatedly shouted, "Cry, cry, why don't you cry, now?"

The *Gazette des Hôpitaux*, of the 19th of February, contains three other cases of the inhalation of the vapor of ether, presented in the wards of Malgaigne. A man, 35

years old, of a nervo-sanguineous temperament, presented, at the internal and inferior part of the right leg, and on the surface of the right internal malleolus, a phlegmonous abscess. Two or three minutes sufficed to put the patient into a state that may be compared to intoxication; indeed intoxication is the word generally used now in Paris in referring to the effects of the ether. After he had answered affirmatively to the questions, whether he felt anything peculiar, whether his sight was troubled? etc., M. Malgaigne, with a bistoury, made on the surface of the abscess and portion of the skin, abundantly supplied with nervous filaments and manifestly inflamed, a longitudinal incision about three or four centimetres in length. The borders of the wound were pressed in order to expel the pus contained in the abscess, during the whole of which procedure the patient gave no sign of pain. The operation being completed, the patient appeared agitated and nervous; his face became red, and his features contracted, the eyelids tightly closed, the muscles in general, and especially those of the face and superior extremities, appeared in a state of abnormal contraction; and from the manner in which, with his eyelids still fast shut, he spit his saliva about in every direction, it was evident that he had lost his reason. This condition of things, however, lasted only two or three minutes, when being interrogated as to what he had felt during the operation, he replied that the pain had been slight, and he knew of no better comparison than to liken it to a slight prick with a sharp instrument. Immediately after his return to consciousness the patient complained of pain, if the lips of the wound were pressed with the finger.

A man, aged 45 years, affected with whitlow of the index finger of the right hand, which made it necessary to remove the member, inhaled the vapors of the ether, at first without effect, owing to the stupidity of the patient who was unable to understand how it should be breathed. However, after he was made to comprehend the mode of inhaling it, it produced intoxication in four minutes, albeit not as perfect as in some other cases, but still to a sufficient degree to diminish the pain so much that he too compared it to a slight prick. The pulse of the patient rose to 88 during the inspiration and attained 92 after the operation.

A girl, about 18 years old, having an affection of the right hand, for which Malgaigne wished to make an incision, was submitted for four minutes to the inhalation of the ether, when the sight became troubled, and an incision of four or five centimetres in length was made on the back of the hand. The sensation experienced was compared by this patient also to that caused by a pricking instrument. What was especially remarkable in this case was the insensibility, a species of stupor, or rather the numbness of the wound, which persisted for a much longer time than in the other patients. For a number of minutes after the operation the fingers or stylet might be plunged into the wound without the patient complaining of any pain. Finally, the sensibility returned, and the patient said she had a burning or scalding sensation on the surface of the wound.

The wards of Prof. Bouillaud, of course, afford no surgical cases, but, as it is fresh in my mind, I may mention a very striking example of the powers of the ether in a young chlorotic girl, who had a very painful and slightly decayed molar tooth, requiring extraction. After inhaling the ether for six minutes her eyelids closed, and she gave every evidence of intoxication. Her mouth was fast shut, however, and the *externe*, who was acting dentist, was quite at a loss how he should introduce the turn-screw; but the patient, while apparently unconscious of everything going on around her, on being told to open her mouth, immediately did so, and although three different attempts were made before the whole of the tooth was extracted, she gave not the slightest manifestation of pain, and on recovering, to the question of whether she had suffered, she replied, "not in the least."

A similar operation, rather more skilfully performed however, attended by similar success, occurred the morning before, while I was in the ward; but so perfectly still was the patient—who on regaining consciousness testified in terms equally strong to the total absence of pain—that I was not aware of it until it was over. I learn from M. Bouillaud, the *chef de clinique*, that several other teeth have been drawn from patients in his wards under the influence of the ether, and in every instance with the most perfect success.

Among the first, if not the first patient whom Velpeau endeavored to intoxicate with the ether, was a man who, after having inhaled it for fifteen minutes without its producing the desired effects—which was attributable, I think, to the fault of the apparatus, an ungainly fixture that had been constructed in the hospital—had his finger removed amid as much pain as this operation usually occasions. Velpeau, on the 22d of January, provided with a much better apparatus, proceeded to remove a tumor about the size of the head of a small fœtus, one similar having already been twice removed, occupying the posterior and superior region of the left thigh. The patient being placed on his belly, had scarcely breathed the ether for four minutes when his head fell upon the pillow, he ceased replying to questions, and his limbs were in a state of complete relaxation. Velpeau removed the tumor in less than two minutes; tying the vessels required a little more time; the patient moved, the apparatus was placed before his mouth, and he awoke no more till the dressing was applied. On being asked if he had suffered, he replied that he had not, but on the contrary had felt exceedingly comfortable, and assured M. Velpeau that this new method was *la bonne methode*.

Larrey, one of the assistants, being struck by the relaxation of the muscles of this individual, suggested that the ether might be of value in the reduction of luxations, by controlling the violent muscular contractions in robust subjects. A short time afterwards he had the pleasure of seeing, in the same room, a dislocated shoulder joint, in a very powerful man, which had resisted all attempts at reduction, easily returned to its proper position when the patient was brought under the influence of the ether. When it is remembered that the subject of the preceding observation had submitted twice to the same operation, previous to the discovery of the powers of the ether, it becomes one of the most conclusive facts in its favor.

The next, a case that occurred in the service of Vidal, at *Du Midi* hospital, seems to prove that the ether may exaggerate instead of lessening the sensibility. The patient, æt. 24 years, laboring under a varicocele, was given the ether without success, owing to the difficulty which he experienced in properly inhaling it. The next day, however, intoxication was produced in the course of twenty minutes, but, unlike any of the former examples, it made the patient very gay, while it rendered his sensibility much more acute.

Another case in which the sensibility was greatly augmented, I saw in the amphitheatre of Prof. Roux. In this instance the patient, who had an abscess of the neck, after breathing the ether for a considerable length of time, suddenly jumped from his chair, commenced screaming, and made various rapid and excited gestures. Being very soon reseated, the professor plunged the bistoury into the abscess, which seemed to give great pain, and the patient expressed himself as having suffered excessively.

It is a question in my mind whether, if this patient had been made to inhale the ether for some moments longer, insensibility and stupor would not have been induced. The opinion of many of the Paris surgeons, as set forth in the medical journals, seems to be opposed to continuing the inspiration of the ether in refractory subjects, that is, in those who become excessively agitated and excited. But in support of the hint thrown out—which, however, I by no means claim as my own—I will refer to several cases in which the sensibility, greatly augmented at first, declined at a later stage, and the patients, sometimes in the liveliest and at other times again in rather furious moods, have, after carrying the intoxication still farther, become lethargic, and in neither gave evidence of pain, nor preserved any recollection of having suffered during the operation. For instance, I witnessed a case of this sort in the service of Velpeau, while I was following a private course at La Charité. The subject, a coachman, had a tumor situated in the region of the parotid gland, where operations are known to be peculiarly painful. The ether soon produced its effects. During the first half of the operation the patient did not move; towards the end, however, he became a good deal agitated, his brows contracted, and he showed a disposition to rise from the table, but the ether was reapplied to his mouth and he again became tranquil. On being asked, when the intoxication had subsided, why he had acted so, he said he had dreamed that he was in a billiard room playing a game with his friends, and getting into a dispute with them, he thought some one in the midst of the quarrel had mounted into the seat of his carriage, which he had left at the door, and was driving off; he was endeavoring to go out and stop him. Another instance of similar bearing I saw on the operating table of Blandin, in a woman of nervous temperament, who, after breathing the ether for a few minutes, became very much excited, attempted to pull the tube from her mouth, and would have succeeded in doing so but for the efforts of two assistants. In a short time she became calm, her head fell back on the pillow, and Blandin striped her knee four times with the actual cautery without her feeling any pain, or even believing, when she recovered her consciousness, that the operation had been performed, until the trace of the iron was shown to her.

M. Guersant, jr., surgeon to the Children's Hospital, among many other cases which I shall not detail, administered the ether to two girls before performing on each a very painful operation. The first, aged 12 years, after inhaling the ether for only two minutes, appeared completely insensible during the whole of the time occupied in the disarticulation of the index finger. Towards the end of the operation, she commenced crying, and, after recovering, said she felt him pulling the flesh, and had wished to cry but found it impossible to do so; and she stated, moreover, that she had very distinctly felt him pinch it twice before he removed the finger. The other patient, likewise 12 years of age, after inspiring the ether two minutes, appeared plunged in a profound stupor. The first incision was made without any manifestation of pain, but M. Guersant had hardly introduced his finger into the wound before the patient screamed violently. She was again made to inhale the vapors of the ether, and the operation was terminated without her giving any other sign of suffering. Her pulse became very small and slow, and remained for quite a long time in this state. The patient had no recollection of having suffered any pain.

On the 19th of January, M. Jobert amputated the right thigh of a young man twenty-four years of age. He became very gay after inhaling the ether for twenty minutes, but soon after showed all the symptoms of intoxication, accompanied with threats and menaces; so violent, indeed, was his excitation, that he was obliged to be held while Jobert removed the limb. The operation being completed, the patient was shown his lost member. With a very long face, he said he "regretted that he could no longer dance as formerly," but, soon brightening up, congratulated himself "that he would no longer be annoyed by a corn on one of his toes." The tying of the vessels gave more pain than the amputation.

M. Gerdy presented to the Academy of Sciences, on the 25th of January, several cases of patients submitted to the vapors of ether, of which the following epitome may be interesting:

A young girl intoxicated, or benumbed, if you think the word preferable, with the ether, lifted her hand to her neck, but uttered no cry, while an incision was being made there; she returned to consciousness a few moments after, laughing most heartily, but did not speak of the incision on her neck; while walking to her bed she staggered a good deal.

A patient operated on ten or twelve days before for a strangulated hernia at the groin, allowed the intestine to escape below the cicatrix of the first operation; Gerdy attempted reduction, but the patient evidently suffered during these efforts, notwithstanding the inhalation of the ether.

Another patient, who was the subject of a cataract for which Gerdy wished to operate by extraction, was made lethargic by the ether. He pricked and pierced the cornea, but the eye became so unsteady that he was obliged to abandon the operation. Gerdy attempted now to depress the cataract, but the eye continued so unsteady that he was compelled to relinquish this also. He then pricked the nose and lips and afterwards the hand of the patient, who on returning to himself, although he remembered very distinctly having been pricked, did not speak of any pain felt in the nose or lips.

In the case of a patient who had been operated upon a short time previously for a fistula in ano, which had not progressed as favorably as could have been desired, the matter having burrowed beneath the integuments, it became necessary to remove a portion of the skin near the anal orifice. The patient inhaled the ether until its characteristic effects were produced, when the operation was performed, and although he suffered much less from this procedure than had been caused by the mere dressings the day before, still he experienced some pain.

A young woman was able to undergo a dilatation of the vagina after breathing the ether, which she could not bear before. She returned to herself soon after, became excessively gay, and was at first incapable either of walking or supporting herself.

I saw M. Boyen amputate the leg of a woman about forty years old, of nervo-sanguineous temperament, who had been rendered insensible by the vapors of the ether. She did not make the slightest movement, and, on the effects passing off, said that she remembered nothing of what had been done to her.

A woman of the same age and temperament had a tumor of the right breast, which I saw Blandin remove while she was under the influence of the ether, without her making any demonstration of suffering, or retaining any recollection of the operation.

Velpeau amputated the leg of a young man previously inebriated by the ether, without his giving the least evidence of suffering. When the third arterial ligature was applied, he cried out, but on returning to himself he did not know why he had done so, for he had felt nothing; and, in accounting for the incoherent sentences uttered by him towards the end of the operation, said they had arisen from his having thought of the unfortunate condition of his family.

On another occasion Velpeau removed a portion of the hand of a young man who had been made insensible by the ether. During the first part of the operation he did not move, but before it was finished he became agitated, made gestures as though he wished to escape, and seemed to suffer pain; but on recovering his sensibility he declared that he had felt nothing, and that his movements were occasioned by his dreaming that he was endeavoring to separate two of his comrades in a quarrel.

About the same time, Velpeau removed a man's eye who was under the influence of the ether, and who said, when the operation was over, that although he was conscious of what was going on, he had not suffered the least pain.

Velpeau opened an abscess in the breast of a young woman who had breathed the ether but six times; this was sufficient to intoxicate her, and when she recovered her consciousness she exclaimed, "Why did you not operate upon me while I was asleep?"

Some time before, a man entered his wards at La Charité with a fractured thigh. The subject was robust, vigorous, and extremely muscular; he was also excessively impressible, was affected with a species of convulsive movement, and it seemed as though it would be difficult to restore the wounded limb to its normal form and strength. After breathing the ether for some minutes he became insensible, although he was somewhat agitated and spoke incoherently, but his muscles were relaxed, yielded to the least traction, and allowed the member to be reduced with the greatest possible facility. When all was finished, the patient said, on recovering his consciousness, that he had not been aware of a single thing that had been done, and only complained of having had an unpleasant dream.

Another patient, a young girl who was subject to nervous attacks, was submitted to the influence of the ether a day or two before it was proposed to remove the nail from her great toe. She soon became insensible, but, on recovering from this state, was taken with a convulsive fit. The next day a new administration was again followed by insensibility, which, as before, was succeeded by convulsive paroxysms; but as the patient complained in no respect, the ether was again administered, and the operation performed without her feeling the slightest pain. On recovering she experienced a long and very intense convulsion. During the operation she raised herself up as if to see what was going on; but she felt nothing, and did not reply to any of the questions put to her. She has since said that she dreamed of assisting at a wedding. M. Velpeau asks, "If the nail had been removed without making her breathe the ether, would she not have had equally, perhaps more violently, these convulsive movements?"

A young physician who experimented upon himself, became insensible very soon without losing his consciousness; he was even able to indicate various experiments that he wished performed, and he stuck pins and a lancet into his flesh without feeling them.

There have been many other examples where the patients continued to see, hear, taste, smell, and perceive what was passing around them, while their sensibility for the time being was totally abolished. This preservation of the ordinary powers of sensation and complete loss of all abnormal feelings, has suggested the following question to the mind of Malgaigne: "Are there in the brain two centres of sensation, one for the normal and the other for those which are abnormal?"

The following is a list of the operations performed in Paris up to the 1st of March, with the names of the hospitals and of the operators:

HOTEL DIEU.

*Prof. Roux.*—Amputation of the leg, 1; amputation of the thigh, 1; amputation of fingers, 3; tumor of the breast, 1; tumor of the face, 1; removal of necrosed bone, 1; lachrymal fistula, 1; fistula in ano, 2; phymosis, 1; incisions, excisions, etc. 30.

*M. Blandin.*—Amputation of one of the bones of the foot, 1; amputation of the breast, 3; fistula in ano, 1; cauterization with the red iron, 5.

*M. Boyer.*—Amputation of the leg, 1; amputation of the finger, 1; removal of a finger nail, 1; moxas, 1.—Total, 56.

LA CHARITÉ HOSPITAL.

*Prof. Velpeau.*—Amputation of the leg, 1; amputation of the breast, 1; sarcocele, 1; partial amputation of the breast, 1; removal of a cancerous tumor on the thigh, 1; removal of a parotid tumor, 1; extirpation of the eye, 1; partial amputation of the hand, 1; puncturing abscess of the breast, 2; fistula in ano, 2; excision of the amygdalæ, 1; removal of the finger nail, 1; reduction of a luxation of the thigh, 1; reduction of a luxation of the elbow, 1; reduction of a fracture of the thigh, 1; injection of iodine into the articulation of the knee, 1.

*M. Gerdy.*—Cataract, 1; amputation of the forearm, 1; removal of polypi of the nose, 1; seton, 1; reduction of hernia, 1; incisions, excisions, etc. 4.—Total, 27.

ST. LOUIS HOSPITAL.

*M. Jobert.*—Amputation of the thigh, 4; amputation of the leg, 1; amputation of the arm, 2; tumor of the breast, 2; various tumors, 3; fistula and fissure of the anus, 2; cauterization with the red iron, 2; reduction of a luxated shoulder, 1; hydrocele, 1; incisions, excisions, etc. 20.

*M. Malgaigne.*—Amputation of the leg, 1; removal of tumors, 2; amputation of fingers and toes, 4; incisions, excisions, etc. 10.—Total, 55.

BEAUJON HOSPITAL.

*M. Langier.*—Amputation of the thigh, 3; amputation of the leg, 1; excision of tumor, 1; strangulated hernia, 1; moxas, 1.

*M. Robert.*—Amputation of the leg, 1; section of the tendo Achillis, 1; reduction of a luxation of the elbow, 1.

*M. Bouvier.*—Section of the tendo Achillis, 1; strabismus, 1.—Total, 12.

HOSPITAL DE LA PITIÉ.

*M. Giraldés.*—Amputation of the thigh, 1; amputation of the big toe, 2; removal of a tumor, 1; hydrocele, 1; removal of a toe nail, 1; incisions, 2; cauterization with the red iron, 4.—Total, 12.

CHILDREN'S HOSPITAL.

*M. Guersant.*—Operation for stone, 1; amputation of the thigh, 1; amputation of the arm, 1; amputation of fingers and toes, 5; fistula in ano, 1; cauterization with the red iron, 1.—Total, 10.

COCHIN HOSPITAL.

*M. Michon.*—Amputation of toe, 1; lithontripsy, 1.—Total 2.

HOSPITAL DE BON LE-COURS.

*M. Denonvilliers.*—Amputation of a finger, 2; excisions, incisions, etc. 18.—Total, 20.

MAISON ROYALE DE SANTÉ.

*M. Monod.*—Amputation of a finger, 1; amputation of a breast, 1; circumcision, 1.—Total, 3.

HOSPITAL DES CLINIQUES.

*M. Voilemier.*—Amputation of a leg, 1; amputation of an arm, 1; other operations, 8.—Total, 10.

DU MIDI HOSPITAL.

*M. Vidal.*—Varicocele, 2; phymosis, 1; strabismus, 1.—Total, 3.—Grand total, 211.

Of these there were of amputation of the thigh, 10; amputation of the leg, 8; amputation of the arm, 4; amputation of the forearm. 1; partial amputation of the foot, 2; partial amputation of the hand, 1; amputation of fingers and toes, 19; total or partial amputation of the breast, 8; extirpation of important tumors in different regions, 12; removal of the eye, 1; sarcocele, 1; strangulated hernia, 1; stone, 1; lithontripsy, 1; hydrocele, 2; varicocele, 2; phymosis, 4; fistula or fissure of the anus, 8; reduction of luxations or fractures, 5; reduction of hernia, 1; removal of nails, 4; extirpation of amygdalæ, 1; polypi of the nasal fossæ, 3; cataract, 1; lachrymal fistula, 1; strabismus, 2; section of tendo Achillis, 2; cauterization with the red iron, 12; application of moxas, 2; opening abscesses, incisions and excisions of various sorts, 91.

Of these the following terminated fatally: Amputation of the thigh, 4; amputation of the leg, 3; amputation of the arm, 2; amputation of the forearm, 1; amputation of a toe, 1; amputation of a finger, 1; amputation of a breast, 2; extirpation of tumors, 3.—Total, 17.

Thus in 10 amputations of the thigh there have been 4 deaths, that is, 2 in 5; while in a report by Malgaigne, made in the *Archives of Medicine*, entitled "*Statistical Studies upon the Results of the Large Operations in the Paris Hospitals*," we find that from the 1st of January, 1836, to the 1st of January, 1841, there were 201 similar operations performed, of which 126, or 3 in 5, had a fatal termination. During these five years, there were 852 amputations of members, from disarticulation of the thigh to amputation of the phalanges, in which the general mortality was 332, or about 2 in 5. Of the 23 operations which represent the amputations of the thigh, arm, and forearm, 10 have died, or about 2 in 5. The statistics of M. Malgaigne give 512 amputations of the same kind, and 281 deaths, or nearly 3 in 5.

I have gone into these details for the twofold purpose of showing the rate of mortality in hospital operations in Paris, and of estimating, if we may, the effect of ether upon this mortality. The number of operations is yet too small to justify any definite conclusions upon the last of these points, though it must be admitted that, as far as they go, they in no wise support the assertion of Magendie, that the influence of the ether upon the constitution is deleterious, and would in many cases dispose the operations to unpleasant terminations.

I saw M. Velpeau yesterday morning, (March 22,) who told me that he had operated upon more than forty patients who had breathed the ether with almost uniform success, that is, with various manifestations, had become more or less intoxicated and insensible to pain; and that he did not believe that he could lay to the charge of the ether the fatal termination in a single instance.

The effects of the ether are as various as the constitutions and temperaments subjected to its influence. In some it produces excessive hilarity, and in others anger; now exciting joy, and again sadness; now inducing the calmest and pleasantest, and again the most troubled and painful slumbers; in some persons giving rise to delightful dreams, and at other times awakening those of an unpleasant character, or steeping the faculties in total unconsciousness; now producing the most profound insensibility, but again affecting the constitution slightly, and in still rarer instances exalting all the sensibilities The lover, under its influence, has often dreamed of his mistress, and very many women have dreamed, while intoxicated with it, that they were in heaven.

But this agent, so varied in its effects, and generally so benign in its action, like all substances of intense power, must be used with certain precautions. Already harm has been done by it; death has been produced by insisting on its application in cases where the subject was violently opposed to its inhalation; and, indeed, in instances where there was no repugnance on the part of the patient to be overcome, unpleasant, and, in one case, fatal pectoral symptoms have been traced to its action. The fatal case occurred in the service of M. Jobert, at the hospital St. Louis, and this surgeon has had the candor to avow his belief, that his patient died of a bronchitis brought on by the ethereal inhalation. Statements of this sort would not have been believed, in France or England, four weeks ago; but the rage for the "*letheon*" is beginning to subside, and the faculty of Paris are now scrutinizing its effects, and endeavoring to determine the circumstances which render its application hazardous. Mr. Liston foresaw and predicted some of the evils which would flow from the indiscriminate use of so powerful an agent. And yet there are those in this city who still profess to doubt whether it has ever done harm in any instance. Velpeau is one of these, and treats all the fears on the subject with derision, holding the agent to be one of equal safety and efficiency. Time and experience must determine the matter. I remember that some cases of phrenitis were published in your Journal two years ago, which were ascribed to the inhalation of ether. If it be capable of doing mischief, we shall not long want evidence of its power, for the hourly administration of it to patients in all conditions, and almost every disease, must soon develop its capacity for evil as well as good.

Since writing the foregoing, I have seen in a Marseilles journal, that M. Pertusio, surgeon of a hospital at Turin, has obtained a very happy result by the aid of etherization in a case of well marked traumatic tetanus. On the 4th of February, a young man was attacked with tetanic symptoms, which by the 13th attained the greatest intensity. It occurred to M. Pertusio that the vapors of ether might avail in the case, and the result of the trial was, almost immediately, the complete relaxation of the muscular rigidity. The tetanic symptoms recurred, however, so soon as the etherization passed away, but were again relieved by its application, which was repeated as many as six times a day. Gradually the symptoms becoming less violent, and recurring at greater intervals, the ether was administered less frequently, and at the end of a week a single etherization each day was found sufficient. The 4th of March, now already eight days since, the patient had not experienced any tetanic symptoms, although the ether was no longer resorted to, and the patient walked about in the ward of the convalescents, retaining merely a slight stiffness in the muscles of the abdomen.

In my next letter I shall have something to report concerning the experience of M. Paul Dubois with ether in cases of difficult labor. For the present, I think you will agree with me, that I have said enough on the subject.

Paris, March 31, 1847.

